# Functioning and equality according to International Classification of Functioning, Disability and Health (ICF) in people with skeletal dysplasia compared to matched control subjects – a cross-sectional survey study

**DOI:** 10.1186/s12891-020-03835-9

**Published:** 2020-12-04

**Authors:** Hanna Hyvönen, Heidi Anttila, Susanna Tallqvist, Minna Muñoz, Sanna Leppäjoki-Tiistola, Antti Teittinen, Outi Mäkitie, Sinikka Hiekkala

**Affiliations:** 1 University of Jyväskylä, Jyväskylä, Finland; 2 Finnish Institute for Health and Welfare; Welfare Department; Aging, Disability and Functioning Unit, Helsinki, Finland; 3 University of Helsinki, Helsinki, Finland; 4 Validia ltd, Helsinki, Finland; 5 Lyhytkasvuiset – Kortväxta ry (Finnish association for people with restricted growth, and for their families), Helsinki, Finland; 6 The Finnish Association of People with Physical Disabilities, Helsinki, Finland; 7 Finnish Association on Intellectual and Developmental Disabilities, Espoo, Finland; 8 Children’s Hospital, University of Helsinki and Helsinki University Hospital, Helsinki, Finland; 9 Folkhälsan Institute of Genetics, Helsinki, Finland

**Keywords:** Skeletal dysplasia, Short stature, Functioning, Accessibility, Equality, Disability, Rare diseases, Orphan diseases

## Abstract

**Background:**

Skeletal dysplasias are rare disorders often leading to severe short stature. This study aimed to gain new comprehensive information about functioning and equality in people affected by skeletal dysplasia compared to matched controls without skeletal dysplasia.

**Methods:**

Functioning was assessed by questionnaire, which was formed by operationalizing International Classification of Functioning, Disability and Health (ICF) core set’s categories into the items according to the ICF linking rules, using primarily Patient Reported Outcomes Measurement Information System PROMIS® - items.

**Results:**

Altogether 80 subjects with skeletal dysplasia and 55 age-, gender- and place of residence -matched controls participated. People with skeletal dysplasia experienced more pain (*p* < 0.001) and the pain interfered more their daily lives (*p* = 0.037) compared to the controls. They had more problems related to musculoskeletal functions and exercise tolerance, difficulties in mobility, used more assistive products and technology and were more affected by climate and seasonal changes (*p* < 0.001). They met challenges in self-care, acquisition of goods and services and household tasks (*p* < 0.001) and in participating in close social relationships, leisure time activities (p < 0.001) and associations and organizational services (*p* = 0.007). They felt less satisfied with remunerative work (*p* = 0.003), felt more inequality (*p* = 0.008), met more negative attitudes of others (*p* < 0.001) and felt having less support given by family and friends (*p* = 0.022). They used more social and health services and experienced more dissatisfaction with those.

**Conclusions:**

Our study indicates that skeletal dysplasias restrict functioning extensively and significantly affect daily living. By building accessible environment and improving equal services, functioning could be improved.

## Background

There are hundreds of medical reasons for short stature. The more than 400 different forms of skeletal dysplasias, rare genetic disorders involving the growth and development of skeletal structures, comprise the largest group [1]. Because of particular population genetics in Finland, diastrophic dysplasia (OMIM #222600) and cartilage-hair hypoplasia (OMIM #250250) are as common as achondroplasia (OMIM #100800), while elsewhere achondroplasia is the most common form and the others very rare [2]. Based on recent studies, the total number of people with skeletal dysplasia in Finland can be estimated to be over 1000 [3, 4]. Skeletal dysplasias vary in their skeletal and extra-skeletal features but are often characterized by short limbs, joint deformities, and normal cognitive development. A major feature in several of these disorders is severe growth retardation leading to disproportionate short stature. This and other potential features have a significant impact on individuals’ functioning [1].

According to prior studies, lower quality of life has been observed in people with skeletal dysplasia compared to the standardized American mean [5], in achondroplasia compared to their unaffected first-degree relatives [6], and in diastrophic dysplasia compared to the control subjects [3]. Additionally, the prevalence of pain is high among people affected with these conditions [3, 5, 7]. Krüger et al. [3] showed that people with diastrophic dysplasia have lower levels of functioning compared with the controls. Johansen et al. [8] reported impaired health status in people with short stature compared to the general population.

Information and scientific data concerning functioning in people with diagnosis of skeletal dysplasia are still very limited and this prevents optimal management and support. In the present study, we used International Classification of Functioning, Disability and Health (ICF) to assess the functioning comprehensively, with its different components: body structures, body functions, activities and participation and environmental factors. The use of ICF-classification provides a scientific basis for studying health and health-related states and outcomes [9]. Because there is no specific ICF core set for people with skeletal dysplasia, we first developed a modified comprehensive musculoskeletal post-acute ICF core set with additional categories to form a questionnaire which content was validated (Anttila H, Tallqvist S, Muñoz M, Leppäjoki-Tiistola S, Mäkitie O, Hiekkala S: Towards an ICF-based self-report questionnaire for people with skeletal dysplasia to study health, functioning, disability and accessibility, submitted). Here we used this newly developed questionnaire to better understand the challenges in functioning faced by people with skeletal dysplasia including environmental factors, such as social, natural and built environment, which is important as functioning can be seen as an interaction between health conditions and contextual factors [9].

The aim of this study was to gain new comprehensive information about the functioning and equality of people with skeletal dysplasia, with the use of ICF and its different components of functioning (body structures, body functions, activities and participation and environmental factors). The results were compared with a cohort of age-, gender- and place of residence -matched controls, to highlight the state of functioning and equality of people with skeletal dysplasia and to have evidence-based baseline data for prospective and interventional studies. Such data in this patient population has been largely lacking.

## Methods

### Study design and setting

This is a quantitative cross-sectional survey conducted in Finland. The survey was approved by The University of Helsinki Ethical Review Board in the Humanities and Social and Behavioral Sciences (statement 1/2016, 13.1.2016).

The survey data was collected in the autumn of 2016 in Finland from people with skeletal dysplasia. Participants were recruited by sending paper inquiries to people with selected skeletal dysplasia diagnosis, identified through the skeletal dysplasia register at Children’s Hospital, University of Helsinki and Helsinki University Hospital. Finland’s population is 5.5 million, and approximately 1.5 million live in the hospital catchment area. However, the patient care for children with skeletal dysplasia is centralized from the whole country to Helsinki University Hospital. Most of the subjects with skeletal dysplasia are included in the register. Altogether paper inquiries were sent to 203 adult people with the diagnosis of diastrophic dysplasia, achondroplasia, or cartilage-hair hypoplasia.

Additionally, there was an open electronic link to the survey, which was available for approximately 2.5 months. Participants were recruited by informing about the electronic survey via social media, patient journals and in the Autumn day of the Finnish patient organization People with Restricted Growth. Subjects receiving the postal questionnaire were also informed about the possibility to respond via the electronic link. The final survey data concerning people with skeletal dysplasia was collected until mid-December 2016.

After the data collection from people with skeletal dysplasia, four age-, gender- and place of residence -control subjects for each participant with skeletal dysplasia were chosen via wide population register of Digital and Population Data Services Agency. The control subjects were coded in a case-control manner, as follows: 1–01, 1–02, 1–03, 1–04, 2–01, 2–02, 2–03, 2–04, 3–01...n-04. The postal questionnaires were sent to the control subjects during February and March 2017. Only the first replier of the control subjects for each person with skeletal dysplasia was chosen, even though there might have been more replies from the four controls.

### Participants

Participants in the present study consisted of people with skeletal dysplasia and their age-, gender- and place of residence matched control subjects. To avoid excessive heterogeneity among the skeletal dysplasia group, only those individuals affected by one of the three most common skeletal dysplasias in Finland were invited: diastrophic dysplasia, achondroplasia, and cartilage-hair hypoplasia.

### Questionnaire

The questionnaire was designed using the comprehensive musculoskeletal post-acute core set of ICF as a framework. All categories were operationalized into the items according to the ICF linking rules [10]. Patient-Reported Outcomes Measurement Information System (PROMIS®) -items, the National Finsote Survey [11], Craig Hospital Inventory of Environmental Factors [12], and Measure of the Quality of the Environment [13] were used as item sources. Face validity was evaluated by an expert panel of four individuals with skeletal dysplasia and content validity of the questionnaire was assessed by thematic interviews of 14 people with skeletal dysplasia (Anttila H, Tallqvist S, Muñoz M, Leppäjoki-Tiistola S, Mäkitie O, Hiekkala S: Towards an ICF-based self-report questionnaire for people with skeletal dysplasia to study health, functioning, disability and accessibility, submitted). Finally, as a result of iterative process, the final survey covered 85 ICF categories and one ICF chapter including 173 ICF-linked items that were grouped to 33 questions, describing the construct of health (body structures and body functions), functioning and disability (body functions and activities & participation), and accessibility (environmental factors). The questionnaire development process is described in detail elsewhere (Anttila H, Tallqvist S, Muñoz M, Leppäjoki-Tiistola S, Mäkitie O, Hiekkala S: Towards an ICF-based self-report questionnaire for people with skeletal dysplasia to study health, functioning, disability and accessibility, submitted).

### Statistical methods

The data was entered into SPSS 26.0 to perform statistical analysis. To begin, all the five-point Likert scale variables (items) were recoded as following: Point 1 indicates “no problems” and point 5 indicates “a lot of problems”. Then sum variables were formed in order to make compact units and to minimize the number of variables. In this process, ICF-classification was used as a frame: sum variables were formed according to ICF blocks (or chapters if the chapter doesn’t contain blocks), which consist of ICF categories with similar content. If variables belonged to the same block (or chapter), a sum variable was computed. All the formed sum variables had five-point Likert scales. The minimum number of variables in one sum variable (in one block or chapter) ranged from 2 to 16 variables. There were also blocks (and chapters) which had only one five-point Likert scale variable, so there was no possibility to form a sum variable and they were analyzed as ordinal scale variables. Internal consistencies of the sum variables were assessed by Cronbach’s alpha, inter-item correlations and item-total statistics. If Cronbach’s alpha of a sum variable was below 0.7 and/or inter-item correlations and item-total statistics indicated that a variable is not part of the scale, following corrections were made: 1) Variable or variables was/were deleted from the sum variable and left out from the analysis. 2) Sum of two variables wasn’t formed and the variables were analyzed as ordinal scale variables. Those corrections were made in order to increase the Cronbach’s alpha above 0.7 and to improve the internal consistencies of the sum variables [14]. Altogether, four variables (items) were left out from the analysis, because they would have decreased sum variable’s internal consistency. The Cronbach’s alphas of the formed sum variables varied from 0.72 to 0.95. Because a great number of variables in a sum variable can increase the alpha [14, 15], it was confirmed, that the sum variables which consisted of over 10 variables, would have had high alfa also in smaller number of variables.

Characteristics of people with skeletal dysplasia and their control group were analyzed by 2-tailed *p*-values and 95% confidence intervals via independent-samples t-test concerning continuous variables. All the nominal scale variables were analyzed by exact 2-sided p-values via crosstabs and Pearson’s chi-square. Mann-Whitney U-test was used for variables with five-point Likert scale and also for continuous sum variables, because they weren’t normally distributed. Normal distribution of variables was tested with Kolmogorov-Smirnov Test for Normality using Lilliefors Significance Correction. A statistical significance level of 0.05 was used. Missing values concerning some single questions in the control group were not included in the analysis.

## Results

Of 203 subjects with skeletal dysplasia who fulfilled the inclusion criteria, seven could not be reached because the address was unknown, or the person had moved to another country or had deceased. Of the remaining 196 individuals with skeletal dysplasia, 80 (40.8%) responded and consented to the study. Majority of the participants (90%) were included in the skeletal dysplasia register while the remaining 10% were reached through the patient organization*.* Of the age-, gender- and place of residence -matched control subjects, 55 participated and replied to the questionnaire. Thirty-nine individuals with diastrophic dysplasia had 27 control subjects, 15 with achondroplasia had 13 control subjects and 26 with cartilage-hair hypoplasia had 15 control subjects (Table 1). Some subjects with skeletal dysplasia lacked a matched control subject; despite this their data were included in the analysis.

**Table 1:** Characteristics of people with skeletal dysplasia compared to their control subjects

Characteristics	SkelDys (***n*** = 80)	Control (***n*** = 55)	***p***-value	95% CI
Diastrophic dysplasia	39	27^a^		
Achondroplasia	15	13^a^		
Cartilage-hair hypoplasia	26	15^a^		
Women, n (%)	65 (81.3)	46 (83.6)	0.821	
men, n (%)	15 (18.8)	9 (16.4)		
Age, years, mean (SD)	43 (14.7)	46 (15.5)	0.266	−8.2...2.3
Height, cm, mean (SD)	127 (11.8)	166 (8.3)	< 0.001	− 42.1...-35.2
Cordage, cm, mean (SD)	107 (18.4)	163 (26.6)	< 0.001	− 65.5...-47.5
Weight, kg, mean (SD)	51 (13.4)	73 (15.1)	< 0.001	−27.5...-17.4
Life situlation, n (%)			0.047	
Student	8 (10.0)	6 (10.9)		
Unemployed	5 (6.3)	7 (12.7)		
Working	37 (46.3)	30 (54.5)		
Disability pensioner	23 (28.7)	4 (7.3)		
Old-age pensioner	7 (8.8)	8 (14.5)		
Cardiovascular diseases, n (%)	18 (22.5)	9 (16.4)	0.512	
Immune system problems/diseases, n (%)	44 (55.0)	23 (41.8)	0.162	
Excretory problems, n (%)	12 (15.0)	5 (9.1)	0.430	
Urinary problems, n (%)	8 (10.0)	5 (9.1)	1.000	
Osteoarthritis, n (%)	54 (67.5)	20 (36.4)	< 0.001	
Joint malalignments, n (%)	63 (78.8)	14 (25.5)	< 0.001	
Fractures, n (%)	4 (5.0)	6 (10.9)	0.316	

Note: *n* number of subjects, *SkelDys* Skeletal dysplasia, ^a^ = Age, gender, and place of residence control subjects for the particular diagnosis. P-values are from Pearson Chi-Square -test’s for nominal scale variables and Independent-Samples T-test for interval scale variables; 95% confidence intervals are from t-tests; Cardiovascular diseases = heart disease, coronary artery disease, hypertension, other cardiovascular disease; Immune system problems/diseases = more infections than normal, asthma or other pulmonary disease, allergies or tumor disease; Excretory problems = some intestinal disease, difficult constipation or long lasting diarrhea; Urinary problems = incontinence or other problem related to urination

### Demographic characteristics, body dimensions and health factors

Demographic characteristics, body dimensions and health factors were collected and are shown in Table 1. The mean age of people with skeletal dysplasia was 43 years and of the control subjects it was 46 years. Those with skeletal dysplasia had lower values in body dimensions as compared with their age, gender and place of residence matched control subjects and the differences were statistically significant (*p* < 0.001). Also, a life situation differed between the two groups (*p* = 0.047): 29 % of people with skeletal dysplasia were in a disability pension, when the same number of the control subjects was 7%. People with skeletal dysplasia had more osteoarthritis and joint malalignments (*p* < 0.001).

### Body functions

People with skeletal dysplasia had lower values in body functions: they had more pain than their control subjects (*p* < 0.001) and the pain interfered more daily living (*p* = 0.037). They had also more problems in muscle functions and movement functions than their control subjects (*p* < 0.001). The most problematic body functions were exercise tolerance functions, which contains ability to work physically over two hours (Z = 7.602, *p* < 0.001), and mobility and stability functions of joints, which includes hypermobility and limited range of motion of joints (Z = 7.546, *p* < 0.001) (Table 2).

**Table 2:** Differences in body functions in people with skeletal dysplasia and their control subjects

Variable	Group	n	Mean rank	Median	Z	*p*-value
Energy and sleep functions ^a^	SkelDys	80	70.86	1.92	−1.423	0.156
	Control	53	61.18	1.67		
Emotional functions ^a^	SkelDys	80	72.49	2.00	−1.835	0.067
	Control	54	60.11	1.67		
Proprioception ^b^	SkelDys	80	69.59	1.00	−1.158	0.359
	Control	55	65.68	1.00		
Sensory functions related to temperature and other stimuli ^b^	SkelDys	80	68.24	1.00	−0.208	0.893
	Control	55	67.65	1.00		
Pain, Sensation of pain ^b^	SkelDys	80	80.86	3.00	−5.304	< 0.001
	Control	53	46.08	1.00		
Pain, Interference of pain ^a^	SkelDys	67	46.46	2.67	−2.082	0.037
	Control	19	33.05	2.00		
Exercise tolerance functions ^b^	SkelDys	80	88.18	3.00	−7.602	< 0.001
	Control	55	38.65	1.00		
Mobility and stability functions of joints ^a^	SkelDys	80	88.20	2.19	−7.546	< 0.001
	Control	54	36.83	1.06		
Muscle functions^a^	SkelDys	80	81.51	2.00	−5.091	< 0.001
	Control	55	48.35	1.00		
Movement functions ^a^	SkelDys	80	82.18	2.00	−5.201	< 0.001
	Control	55	47.37	1.00		
Protective functions of the skin ^b^	SkelDys	80	67.56	1.00	−0.171	0.866
	Control	55	68.64	2.00		

Note: Variables = Modified ICF-block or chapter -items; SkelDys = people with skeletal dysplasia; Number of n is smaller in the variable “interference of pain”, because it illustrates those replies with pain from mild to extremely hard pain (no replies from people with no pain); ^a^ = a sum variable (interval scale variable); ^b^ = an ordinal scale variable; The bigger the mean rank and the median is, the more the group had problems in the variable; Mann-Whitney U-test for interval and ordinal scale variables

### Activities and participation

People with skeletal dysplasia experienced more restrictions in activities and participation: changing and maintaining body position, carrying, moving and handling objects, walking and moving, using public transportation, self-care, household tasks, taking part to family and informal social relationships and spending time within recreation and leisure time activities (*p* < 0.001). They also experienced more dissatisfaction in remunerative work (*p* = 0.003) and inequality (*p* = 0.008) than their control subjects (Table 3). Acquisition of goods and services was the most challenging activity for the people with skeletal dysplasia, whereas it was the easiest activity for the controls (Z = 9.386, *p* < 0.001).

**Table 3:** Differences in activities and participation in people with skeletal dysplasia and the control subjects

Variable	Group	n	Mean rank	Median	Z	*p*-value
Acquiring skills ^a^	SkelDys	80	69.69	1.00	−1.113	0.281
	Control	55	65.54	1.00		
Making decisions ^a^	SkelDys	80	69.89	1.00	−1.653	0.141
	Control	55	65.25	1.00		
Carrying out daily routine and handling other psychological demands ^a^	SkelDys	80	70.51	2.00	−1.111	0.268
	Control	54	63.04	1.67		
Understanding spoken messages ^b^	SkelDys	80	69.38	1.00	−1.677	0.145
	Control	55	66.00	1.00		
Changing and maintaining body position ^a^	SkelDys	80	85.40	1.83	−6.418	< 0.001
	Control	55	42.69	1.00		
Carrying, moving, and handling objects ^a^	SkelDys	80	90.38	1.86	−8.250	< 0.001
	Control	55	35.45	1.00		
Walking and moving ^a^	SkelDys	80	89.93	2.27	−7.982	< 0.001
	Control	55	36.10	1.00		
Using public transportation ^a^	SkelDys	80	88.09	3.50	−7.631	< 0.001
	Control	55	38.77	1.00		
Self-care ^a^	SkelDys	80	83.38	1.43	−6.107	< 0.001
	Control	55	45.64	1.00		
Acquisition of goods and services ^a^	SkelDys	80	93.66	3.00	−9.386	< 0.001
	Control	55	30.68	1.00		
Household tasks ^a^	SkelDys	80	86.86	1.86	−6.811	< 0.001
	Control	55	40.56	1.14		
Family and informal social relationships ^a^	SkelDys	80	78.79	2.00	−3.887	< 0.001
	Control	55	52.31	1.40		
Remunerative work ^a^	SkelDys	80	76.03	2.00	−2.967	0.003
	Control	55	56.33	1.00		
Recreation and leisure time ^b^	SkelDys	80	78.33	2.00	−3.881	< 0.001
	Control	55	52.97	1.00		
Equality b	SkelDys	80	74.78	2.00	−2.665	0.007
	Control	55	58.14	2.00		

Note: Variables = Modified ICF-block or chapter-items; SkelDys = people with skeletal dysplasia; ^a^ = a sum variable (interval scale variable); ^b^ = an ordinal scale variable; The bigger the mean rank and the median is, the more the group had problems in the variable. Used statistical analysis: Mann-Whitney U-test for interval and ordinal scale variables

### Environmental factors

People with skeletal dysplasia met more challenges with environmental factors: they used more assistive products and technology and they were affected more by climate and season-related chances (*p* < 0.001). They got less support given by family and friends (*p* = 0.22) and had more barriers in participating in associational and organizational services (*p* = 0.007) than their control subjects. The biggest difference between the two groups were in attitudes of others, as people with skeletal dysplasia met more negative attitudes of others, like discrimination, avoidance, and underestimation of their needs (Z = 6.771, *p* < 0.001). Of people with skeletal dysplasia, 56.3% had a need to house modifications and 65.3% to car modifications and the differences were statistically significant compared to their control subjects (*p* < 0.001) (Table 4).

**Table 4:** Differences in environmental factors in people with skeletal dysplasia and control subjects

Variable	Group	n (%)	Mean rank	Median	Z	*p*-value
Products and technology ^a^	SkelDys	80	85.13	1.75	−6.363	< 0.001
	Control	55	43.09	1.00		
Climate and season-related changes ^a^	SkelDys	80	84.66	2.50	−6.085	< 0.001
	Control	55	43.76	1.00		
Support given by family and friends ^a^	SkelDys	80	74.31	1.80	−2.288	0.022
	Control	55	58.83	1.20		
Attitudes of others ^a^	SkelDys	80	85.98	1.92	−6.771	< 0.001
	Control	54	40.13	1.00		
Associations and organizational services ^b^	SkelDys	80	75.14	2.00	−2.695	0.007
	Control	55	57.62	1.00		
Modifications to house entrance ^c^	SkelDys	29 (36.3)				< 0.001
	Control	0 (0.0)				
Modifications to house ^c^	SkelDys	45 (56.3)				< 0.001
	Control	1 (1.8)				
Modifications to car ^cd^	SkelDys	49 (65.3)				< 0.001
	Control	6 (13.0)				

Note: Variables = Modified ICF-block or chapter -items; SkelDys = people with skeletal dysplasia; ^a^ = a sum variable (interval scale variable); ^b^ = an ordinal scale variable; ^c^ = a nominal scale variable; ^d^ = Of those who use a car; Used statistical analysis: Mann-Whitney U-test for interval and ordinal scale variables and Pearson Chi-Square -test for nominal scale variables. The bigger the mean rank and the median is, the more the group had problems in the variable

**Fig. 1:**
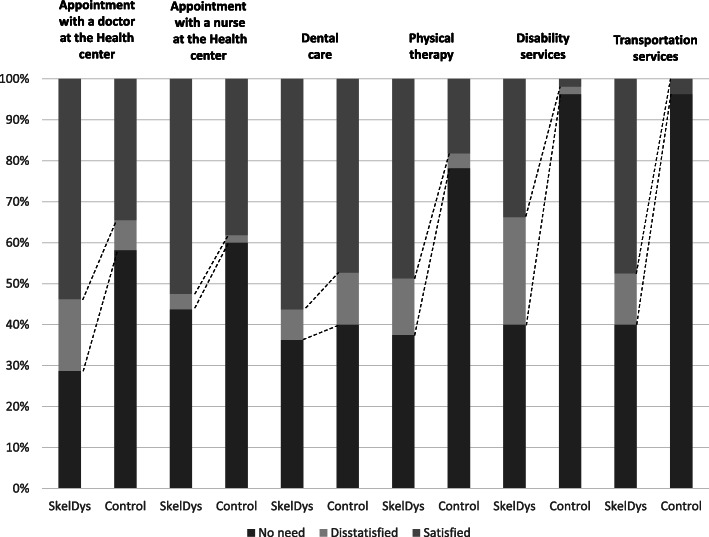
The use of and satisfaction to social and health services. SkelDys = people with skeletal dysplasia (*n* = 80); Control = age-, gender and place of residence control subjects (*n* = 55); Dissatisfied includes people who were dissatisfied with the service or were not allowed to have service

One item in the questionnaire clarified the use of and satisfaction to 27 Finnish social and health services. The most used services (with at least 50% use) by the people with skeletal dysplasia were appointment with a doctor and nurse at the health care center, dental care, physical therapy, disability services and transportation services. The biggest differences between people with skeletal dysplasia and their control subjects in the use of and satisfaction to services were in physical therapy, disability and transportation services (*p* < 0.001) and in doctor appointment at the health care center (*p* = 0.002). People with skeletal dysplasia needed more appointments with doctor, more physical therapy, disability services and transportation services than the control subjects. Of those individuals who had been in a doctor’s appointment, 24.5% of people with skeletal dysplasia were dissatisfied, when the same number of the control subjects were 17.4%. Twenty-six percent of people with skeletal dysplasia were dissatisfied with disability services, which mean that 44% of those users of disability services were dissatisfied (Fig. 1).

## Discussion

This study created new information concerning functioning and equality of people with skeletal dysplasia in relation to their matched control subjects. The results showed that people with skeletal dysplasia have significantly more problems in all ICF-components than their control subjects: in body functions, activities and participation and environmental factors. Generally, results were in line with the previous studies: Krüger et al. [3] found that people with diastrophic dysplasia had lower levels of functioning compared with the controls and Johansen et al. [8] reported impaired health status in people with short stature compared to the general population. Previous studies have reported also that prevalence of pain is high among the people with these diagnoses [3, 5, 7]. However, in the present study, functioning was studied in wider aspect as the environmental factor -component of ICF classification were taken into account and the three most common skeletal dysplasias were represented.

According to the results of this study, people with skeletal dysplasia experienced limitations in body functions, in activities and participation and in environmental factors. The most problematic body functions were exercise tolerance functions and mobility and stability functions of joints. They also experienced more pain. Compared to the controls, people with skeletal dysplasia had more problems in activities and participation -related items of which acquisition of goods and services was the most challenging activity, whereas it was the easiest activity for the controls. These results appear to be reasonable, because due to short stature, people with skeletal dysplasia have more difficulties in reaching. In public spaces, e.g. by ATMs and payment terminals, in small shops, restaurants and gasoline stations, accessibility is often poor. Also due to musculoskeletal problems and restrictions in body function-related items, moving can be more uncomfortable.

This study indicates that people with skeletal dysplasia had more challenges with the environmental factors, such as natural, built and social environment. This is important as the environmental factors are interacting dynamically with a person’s health condition and functioning [9]. The biggest difference in the environmental factors between the two groups was in the attitudes of others: people with skeletal dysplasia faced more negative attitudes from others, such as discrimination, avoidance and underestimation of their needs, including also attitudes of health care providers. This observation is in line with the Dhiman et al. [5] who found that 60% of people with skeletal dysplasia felt treated differently by medical professionals due to their height. Further, our study showed that they used more assistive products and technology, which is not surprising as they have more difficulties with body functions. Difficulties were met also regarding climate and season-related changes and taking part to associations and organizational services: this indicates that more attentions should be paid to removing environmental barriers.

People with skeletal dysplasia used more health and social services than their controls which, to our consideration, seems to be reasonable due to musculoskeletal diseases and pain. They also were more dissatisfied with the appointments with the health care specialist. It is possible that not enough attention has been paid on pain management as people with skeletal dysplasia experienced more pain than the control subjects according to this study and supported by earlier studies [3, 5, 7].

It is worth of noticing, that people with skeletal dysplasia experienced themselves more inequal compared to the control subjects. Also, the differences in several items of perceived functioning between people in these two groups indicate the inequal situation. This indicates clearly that the United Nations Convention on the Rights of Persons with Disabilities isn’t to be fulfilled in the Finnish welfare society. Social and health care services should be improved to be equally available and to meet the specific and demonstrated needs of people with skeletal dysplasia. More attention should be paid to pain experience, and better access to rehabilitation and different supporting services might be necessary to enable better functioning and equality for people with skeletal dysplasia. At this point, there is a significant gap between the current situation and the desired future. Although similar problems have been identified in previous studies, the services still seem to be inadequate. With the different solutions of a built environment and by improving services, equal possibilities to move, perform daily duties and to take part to different activities and services can be provided.

This study had some limitations. The questionnaire was prepared to the present study, and it was not used before. However, the relevance was confirmed and comprehensiveness of the content was validated by people with skeletal dysplasias, as well as its understandability and feasibility were tested and accepted by them (Anttila H, Tallqvist S, Muñoz M, Leppäjoki-Tiistola S, Mäkitie O, Hiekkala S: Towards an ICF-based self-report questionnaire for people with skeletal dysplasia to study health, functioning, disability and accessibility, submitted). The response rate was 40,8%, which was satisfactory while the number of respondents was 80 and all skeletal dysplasias are rare diseases. The present study was restricted only to three most common diagnoses of skeletal dysplasias to avoid heterogeneity. The number of control subjects was smaller than that of people with skeletal dysplasia, which might cause some bias. Most of the subjects with skeletal dysplasia were women, and although the gender distribution was similar in the control group, it is possible that the observations do not fully reflect situation among men with skeletal dysplasia. In statistical analysis, ICF-classification was a good tool to formulate internally consistent sum variables: the contents of the ICF-blocks/ chapters were homogenous but wide enough, to form compact units and to decrease the great number of items, and allowed results to be presented more compactly. Still, some items needed to be dropped out of the statistical analysis if they decreased the internal consistency of the sum variable. Due to its cross-sectional nature, our study does not provide a comprehensive view of the causes leading to the subjects’ medical, social and psychological situation or the causes for the observed differences between the two groups. These need to be evaluated in future studies with more detailed clinical and questionnaire data. For example, more research about the reasons behind the dissatisfaction of people with skeletal dysplasia with health and social services and remunerative work might be needed in the future to improve equality of the people with skeletal dysplasia. The obtained data can be used as a baseline for future longitudinal or intervention studies, to evaluate how potential improvements have affected people living with skeletal dysplasia.

The results of the study can be well generalized to people with skeletal dysplasia in Finland as the sample of the respondents was encompassing due to national register. Clinical features of the particular skeletal dysplasia diagnoses are the same all over the world, although social culture and physical environment with its barriers and accessibility features can vary between countries.

## Conclusions

This study gave comprehensive information about functioning, disability, and challenges in accessibility and equality in people with skeletal dysplasia: skeletal dysplasia restricts functioning extensively affecting individuals’ daily living and causing inequality. This study has a clear clinical implication: By providing and building accessible services and environmental solutions, their functioning and equality could be improved.

## Data Availability

The datasets generated and analyzed during the present study are not publicly available due to individual privacy but are available as anonymous from the corresponding author on reasonable request and from Finnish Social Science Data Archive. Based on the HealthMeasures Terms of use, a clean copy of the questionnaire is not allowed to be included in the manuscript because it includes PROMIS questions. All the PROMIS questions are available in English at www.healthmeasures.net.
